# Insulin Like Growth Factor 2 Expression in the Rat Brain Both in Basal Condition and following Learning Predominantly Derives from the Maternal Allele

**DOI:** 10.1371/journal.pone.0141078

**Published:** 2015-10-23

**Authors:** Xiaojing Ye, Amy Kohtz, Gabriella Pollonini, Andrea Riccio, Cristina M. Alberini

**Affiliations:** 1 Center for Neural Science, New York University, New York, NY, 10003, United States of America; 2 Graduate Program in Psychology, University at Albany–SUNY, Albany, NY, 12222, United States of America; 3 DiSTABiF, Second University of Naples, Caserta 81100, Italy; 4 Institute of Genetics and Biophysics A. Buzzati-Traverso, CNR, Naples, 80131, Italy; CNRS, FRANCE

## Abstract

Insulin like growth factor 2 (*Igf2*) is known as a maternally imprinted gene involved in growth and development. Recently, *Igf2* was found to also be regulated and required in the adult rat hippocampus for long-term memory formation, raising the question of its allelic regulation in adult brain regions following experience and in cognitive processes. We show that, in adult rats, *Igf2* is abundantly expressed in brain regions involved in cognitive functions, like hippocampus and prefrontal cortex, compared to the peripheral tissues. In contrast to its maternal imprinting in peripheral tissues, *Igf2* is mainly expressed from the maternal allele in these brain regions. The training-dependent increase in *Igf2* expression derives proportionally from both parental alleles, and, hence, is mostly maternal. Thus, *Igf2* parental expression in the adult rat brain does not follow the imprinting rules found in peripheral tissues, suggesting differential expression regulation and functions of imprinted genes in the brain.

## Introduction

In most tissue of mouse, rat and human, the insulin like growth factor 2 gene (*Igf2* or *IgfII*) has been found to be maternally imprinted, hence expressed from the paternal allele starting from early developmental stages [1–7].

Imprinting is generally well conserved across mammals, especially between rodents and human, but the reasons for this regulation of expression are still poorly understood [8]. Imprinted genes play an important role in growth and development and their dysfunction is associated with developmental, brain disorders or cancer [9, 10]. In humans, loss of imprinting or uniparental dysomy of imprinted genes contributes to diseases such as Prader-Willi, Angelman, and Beckwith-Wiedemann syndromes, all of which present cognitive dysfunctions [11]. Thus, it is important that the regulation of imprinted genes, particularly in the brain, be better understood.

In line with its role in development, *Igf2* expression is higher during development and significantly decreases in adulthood [12–14]. However, interestingly, compared to most tissues, *Igf2* expression remains relatively elevated in the adult brain [12, 15], where its function has only recently started to be unraveled. Chen et al. (2011) showed that IGF2 in the adult rat hippocampus is upregulated after learning and required for memory consolidation [16]. Moreover, administration of recombinant IGF2 with learning or memory reactivation significantly enhances several types of hippocampal-dependent memories [16, 17] and fear extinction [17, 18], suggesting that IGF2 acts as a potent memory or cognitive enhancer. Furthermore, increasing IGF2 levels in the hippocampus of Alzheimer’s disease model mice rescues cognitive, cellular and synaptic impairments, suggesting that IGF2 might represent a target for regulating cognitive functions in neuropsychiatric diseases [19, 20].

The imprinting status of *Igf2* in most adult brain regions remains largely unknown [3]. However, some studies have suggested that the paternal imprinting in this tissue is not complete, as the maternal expression of *Igf2* has been reported in the human pons [21]. Thus, here we investigated the allelic expression of *Igf2* in adult rat tissues, particularly in brain regions critical for memory formation and storage, and whether learning and/or long-term memory formation alters *Igf2* allelic expression.

## Materials and Methods

### Animals

Brown Norway (BN) and Fischer 344 (F) rats used in the studies were obtained from Charles River Laboratories. F1 offspring of F (dam) x BN (sire) rats and the reciprocal BN (dam) x F (sire) rats were bred in-house. Unless specified, F1 offspring from both crosses and both genders were used. *Igf2* expression and imprinting analyses in adult animals was done in 3–4 month-old rats. *Igf2* expression and imprinting analyses during postnatal development was done at postnatal day (PND) 1, 7, 17 (2–3 days after eye opening) and 24 (3 days after weaning). All the animals were housed in the New York University animal facility and maintained on a 12hr light/dark cycle. Pups remained with their mothers until weaning (PND 21), after which they were housed 2 per cage. Experiments were performed during the light cycle. All rats were allowed *ad libitum* access to food and water. All protocols complied with the National Institutes of Health Guide for the Care and Use of Laboratory Animals and were approved by the New York University Animal Welfare Committee.

### Isolation of DNA and RNA for *Igf2* expression analysis

Rats were euthanized by decapitation. Their tissues were quickly removed and snap-frozen by pre-chilled 2-methylbutane on dry ice and stored at -80°C until further use. Blood samples were collected at the time of decapitation and immediately frozen on dry ice. Brain regions, including dorsal hippocampus (dHC), anterior cingulate cortex (ACC), prelimbic/infralimic cortex (PrL/IL) and basolateral amygdala (BLA) were punched out in a cryostat via a neuropunch (19 gauge, Fine Science Tools, Foster City, CA) as described previously [22] and immediately submerged into Qiazol (Qiagen, Venlo, Netherlands). We carefully avoided contamination of choroid plexus or leptomeninges, where *Igf2* mRNA is highly expressed, by avoiding taking the tissues surrounding the ventricles and immediately adjacent to leptomeninges. Choroid plexus (CP) was collected from the lateral and third ventricles. Total RNA was isolated using the RNAeasy Plus Universal Kit following the manufacture’s protocol (Qiagen, Venlo, Netherlands) and 250 ng of RNA was reverse-transcribed using QuantiTect Reverse Transcription Kit (Qiagen, Venlo, Netherlands). Genomic DNA (gDNA) was isolated from the tails using DNAeasy Blood & Tissue Kit following the manufacture’s protocol (Qiagen, Venlo, Netherlands).

### PCR analyses

For analysis of allele-specific *Igf2* expression based on size differences in PCR products, the *Igf2* 3’untranslated region (3’UTR) around a (CA)_n_ repeat region was amplified by PCR (forward primer: 5’-CTGTGAACAACAATAGCCGC-3’ and reverse primer: 5’-TCCAATCGAAGTTGCTCAGC-3’) using the HotStarTaq Plus Master Mix Kit (Qiagen, Venlo, Netherlands). PCR amplification was performed as follows: an initial denaturation step at 95°C for 5min, followed by 40 cycles at 94°C for 30sec, 60°C for 30sec, 72°C for 20sec, and a final extension step at 72°C for 10min. PCR products were electrophoresed in 2% agarose gel. Because the expression of *Igf2* is much higher in the brain and heart compared to other tissues, in order to detect the PCR products in the gel, 10 fold less cDNA concentration from the brain regions and the heart, as compared to other peripheral tissues, were used as templates. Given the existence of a polymorphism between BN rats and F rats in the amplified region, a 310 bp PCR product was amplified from the allele inherited from BN rats and a 337 bp PCR product was amplified from the allele inherited from F rats.

Quantitative real-time PCR (qPCR) analysis was performed using iQ5 Real-Time PCR System or CFX96 Touch Real-Time PCR Detection System (Bio-Rad, Hercules, CA) with iQ SYBR Green Supermix (Bio-Rad, Hercules, CA). Primers used for qPCR analysis include: total *Igf2* primer set I (forward: 5’-ATGTCACCCATGTCACCAAG-3’, reverse: 5’-GGCTTGTGCCAATTAGGTTCT-3′), total *Igf2* primer set II (forward: 5’-CGTGGCATCGTGGAAGAGT-3’, reverse: 5’-ACGTCCCTCTCGGACTTGG-3’), BN-specific *Igf2* primers (forward: 5’-TGTCACCAAGGGGCTGGGTA-3’, reverse: 5’-GATTTTTGGGACTGGGGGCT-3’), F-specific *Igf2* primers (forward: 5’-AATGTCACCCATGTCACCCAT-3’, reverse: 5’-GGCTTGTGCCAATTAGGTTCT-3’), 18S primers (Qiagen, Venlo, Netherlands, Cat.#: QT00199374). 2.5 ng of cDNA or genomic DNA (gDNA) was amplified using the following conditions: an initial denaturation step at 95°C for 5min, followed by 40 cycles at 95°C for 15sec, 59°C for 30sec and 72°C for 20sec. Triplicates of each sample were analyzed by qPCR, and the averaged cycle threshold (Ct) value was used for quantification using the relative quantification method (see Critical Factors for Successful Real-Time PCR by Qiagen, Venlo, Netherlands). The standard curve for each primer was generated from the amplification of a serial dilution of gDNAs from F1 offspring of F x BN and BN x F rats, which was used to calculate primer amplification efficiency and to determine the relative *Igf2* mRNA concentration in the samples. gDNA of F1 offspring of FxBN rats (which contains equal amount of parental *Igf2* gene) was run on each plate for calibration of allele-specific *Igf2* expression. Samples that contain Ct value greater than 36 were excluded from analysis, considering the loss in qPCR calculation accuracy at this level [23, 24]. The level of total *Igf2* (maternal and paternal) in each sample was normalized to the level of 18S of the same sample, and shown as a ratio to a control sample as detailed in each figure.

For qPCR analysis of *Igf2* expressed from different promoters, the following primers were used: P1-specific *Igf2* primers (forward: 5’-CCACTTCTGCAGCTCTC-3’, reverse: 5’-AAGCACCAACATCGACTTC-3’), P2-specific *Igf2* primers (forward: 5’- CGCTGTTCGGTTTGCAT-3’, reverse: 5’-CGAAGGCCAAAGAGATGAG-3’), P3-specific *Igf2* primers (forward: 5’-GAGAACCTTCCAGCCTTT-3’, reverse: 5’-GAGATGAGAAGCACCAACA-3’) and 18S primers. 6.25ng cDNA was amplified with iQ SYBR Green Supermix using the following conditions: an initial denaturation step at 95°C for 3min, followed by 40 cycles at 95°C for 15sec, 60°C for 30sec and 72°C for 20sec. Triplicates of each sample were analyzed by qPCR, and the averaged cycle threshold (Ct) value was used for quantification using the following equation: Relative expression = 2 –^deltaCt^, deltaCt = (Ct of *Igf2* mRNA variant)–(Ct of 18S rRNA). A template containing 1:1:1 amount of purified PCR products generated from each primer set was also loaded on the qPCR plate to calculate the amplification efficiency for each promoter-specific primer set and used for calibration of the Ct values.

### Pyrosequencing analysis of allelic *Igf2* expression

The pyrosequencing assay was designed and carried out by EpigenDx (Hopkinton, MA), based on a SNP of C_7_/C_8_ in the 5’UTR of *Igf2* expressed from P2 identified by sequencing. cDNA samples from BN and F rat brains mixed at different ratios were used for validation of the assay. cDNAs extracts from a variety tissues of F1 offspring of F x BN and BN x F rats were then analyzed by the pyrosequencing assay, which gave the percentage of C_7_ *vs*. C_8_ allele in the *Igf2* transcript.

### Western Blot analysis

Western blot analyses were carried out as previously described [16, 17]. 20 μg of each protein sample were resolved using 15% SDS-PAGE gel and transferred to an Immobilon-FL membrane (Millipore, Billerica, MA) by electroblotting. The membrane was sequentially blotted with anti-IGF-2 antibody (1:1000, Abcam, Cambridge, UK, Cat#: ab9574) and anti-rabbit IRDye800CW (1:20,000, Li-Cor, Lincoln, NE). A duplicated SDS-PAGE gel was run and stained by Blue Coomassie staining, which served as loading reference for each sample. Staining for housekeeping genes could not be used, as they are expressed at different levels in different tissues. The membrane and Coomassie-stained gels were scanned on the LiCor Odyssey Imager under non-saturating conditions.

### Inhibitory avoidance (IA)

All the rats were handled for 3min per day for 5 days before IA training and then randomly assigned to either the naïve or the trained group. IA was carried out as described previously [16]. The IA chamber consisted of a rectangular-shaped box, divided into a safe compartment that was white and illuminated and a shock compartment that was black and dark (Model ENV-010MC, Med Associates, St. Albans, Vermont). Foot shock was delivered through the grid floor of the dark chamber via a constant current scrambler circuit. The IA box was located in a sound-attenuated room illuminated by red light. During the training session, the rat was placed in the safe compartment. After 10 sec, the door separating the two compartments was automatically opened, allowing the rat access to enter the shock (dark) compartment. The automatic door closed 1 sec after the rat entered the shock compartment, and a brief foot shock (0.9 mA for 2 sec) was delivered. Ten sec after the footshock, the rat was returned to the home cage. Controls consisted of littermates that remained in their home cage (naïve). The rats were euthanized 20hrs after IA training, or at the corresponding time and day for the naïve rats. This is the time point following training at which our prior studies showed an increase in *Igf2* mRNA expression in hippocampus [16].

### DNA methylation analysis

Liver, CP, dHC (from naïve and trained rats) and ACC (from naïve and trained rats) were rapidly dissected out after decapitation, immediately frozen on dry ice and stored at -80°C. The methylation levels of the selected 15 CpG sites in the *Igf2*/H19 imprinting control region (ICR; chr1:222645310–222645423, -4433 to -4530 from H19 TSS), 19 CpG sites in the *Igf2* differentially methylated region 2 (DMR2; chr1: 222726044–222725835, 3492 to 3701 from *Igf2* TSS) and 11 CpG sites in the *Igf2* promoter 2 region (P2; chr1: 222731999–222731819, -2464 to -2284 from *Igf2* TSS) were determined by pyrosequencing, serviced by EpigenDx (Hopkinton, MA). Briefly, 500 ng of gDNA was bisulfite treated using a proprietary bisulfite salt solution (EZ DNA Methylation Kit, Zymo Research, Irvine, CA) and purified using Zymogen DNA columns (Zymo Research, Irvine, CA). 5ng of the converted DNA was used in a PCR reaction to amplify the target region. One of the PCR primers was biotinylated. Single-stranded biotinylated PCR products were purified using the Pyrosequencing Vacuum Prep Tool (Qiagen, Venlo, Netherlands). 10 μL of PCR products were sequenced by the Pyrosequencing PSQ96 HS System (Biotage AB, Uppsala, Sweden) following the manufacture’s instructions. The methylation status of each locus was analyzed individually as a T/C SNP using QCpG software (Qiagen, Venlo, Netherlands). Duplicate samples of each tissue/condition were analyzed, and the averaged % methylation at each CpG site was used for quantification.

### Statistical analyses

Data are reported as mean ± SEM. For biochemical studies, power calculation of one-way ANOVA analyzed by G*Power software indicated a sample size of 4–5 rats per group was necessary to achieve power of 0.8 and an error probability of 0.05. Statistical analyses were designed using the assumption of normal distribution and similar variance among groups. For multi-group comparison, one-way ANOVA followed by Newman-Keuls *post hoc* tests for selected groups were used. For paired comparisons, Student’s t-test was used. All analyses are two-tailed.

## Results and Discussion

**Fig 1:**
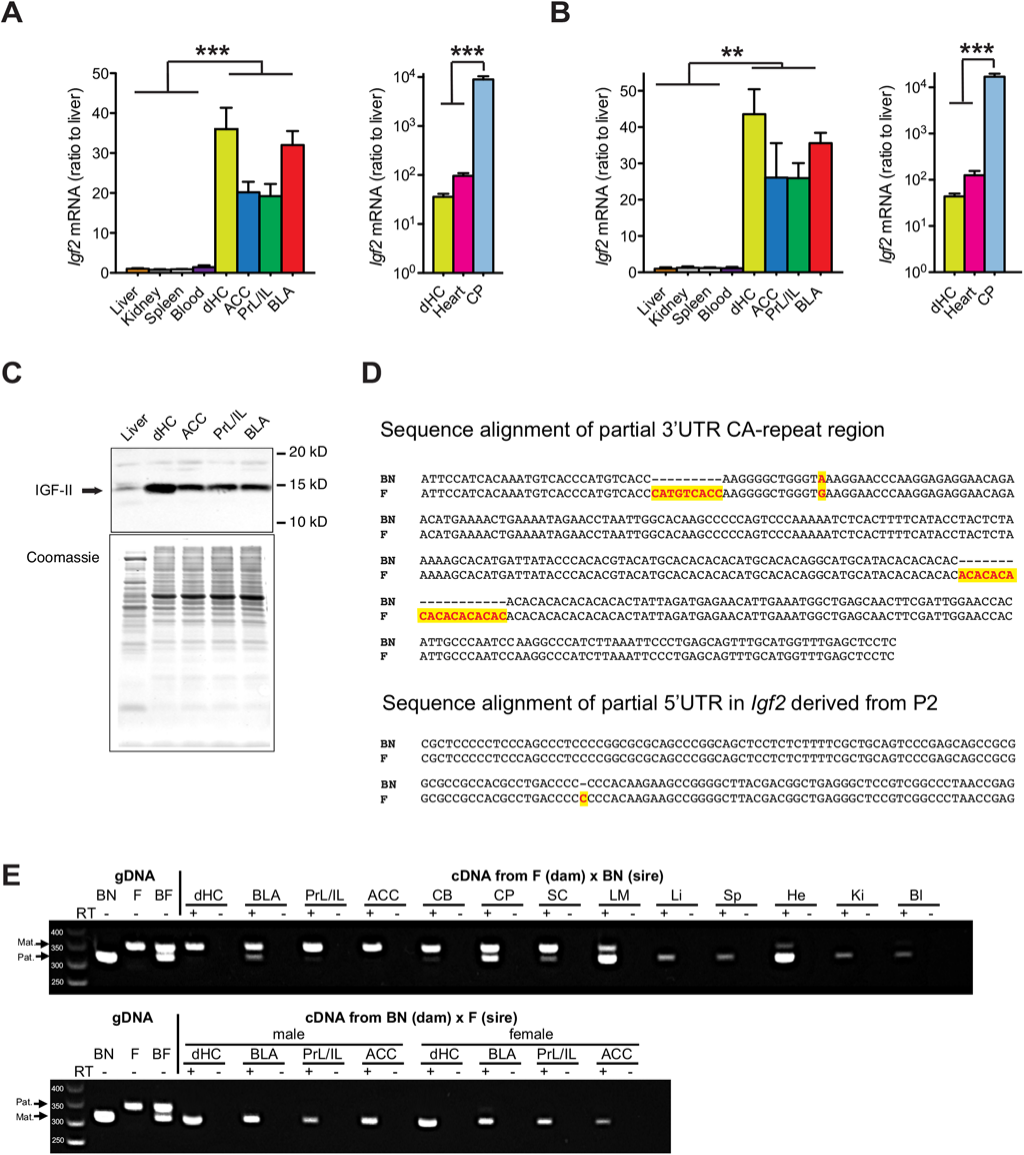
The expression of *Igf2* is significantly and dramatically higher in adult rat brain regions compared to peripheral tissues, and is mainly expressed from the maternal allele in the brain and from the paternal allele in peripheral tissues. (A-B) Quantitative real-time PCR analysis of the relative *Igf2* mRNA levels in liver, kidney, spleen, blood, heart, dorsal hippocampus (dHC), anterior cingulated cortex (ACC), prelimbic/infralimbic (PrL/IL), basolateral amygdala (BLA) and chorioid plexus (CP) using two independent pairs of primers (n = 3–9; for primer set I: F_(9, 59)_ = 39.08, *P* < 0.0001; for primer set II: F_(9, 51)_ = 103.4, *P* < 0.0001). In each sample, *Igf2* mRNA level was normalized to the 18S RNA level. Data are expressed as mean ± SEM of the ratio of *Igf2* mRNA in the liver. **P<0.01, ***P<0.001 (C) Western blot analysis of IGF-2 protein levels in the dHC, ACC, PrL/IL and BLA, compared to that in liver (top panel). A parallel, duplicated gel was used for coomassie staining to reveal protein loading in each sample (bottom panel). This experiment was repeated on 3 rats and showed similar data. (D) DNA sequence alignment of the (CA)_n_ repeat region of *Igf2* 3’UTR and of 5’UTR derived from P2 between Brown Norway (BN) and Fischer (F) rats. Genetic polymorphisms are highlighted. (E) cDNAs obtained from different tissues from adult F1 offspring of BN (dam) x F (sire) (BF) and F (dam) x BN (sire) (FB) rats as well as genomic DNA from BN, F, and their F1 rats were used as templates for PCR amplification of the (CA)_n_ repeat region. The PCR products were resolved by electrophoresis on 2% agarose gel. The 337 bp band represents the allele from the F rats, whereas the 310 bp band represents the allele from the BN rats. PCR products from reactions run in parallel in the absence of reverse transcriptase (RT-) were included to show that the observed bands of RT-PCR products were not due to gDNA contamination. Abbreviations of different tissues: dHC–dorsal hippocampus, BLA–basolateral amygdala, PrL/IL–prelimbic/infralimbic cortex, ACC–anterior cingulate cortex, CB–cerebellum, CP–choroid plexus, SC–spinal cord, LM–leptomeninges, Li–liver, Sp–spleen, He–heart, Ki-kidney, Bl-blood. These data were confirmed by 2 experimenters in a total of 5 independent experiments.

Quantitative real-time PCR (qPCR) analyses carried out on RNA extracted from liver, kidney, spleen, blood, heart, dorsal hippocampus (dHC), anterior cingulate cortex (ACC), prelimbic/infralimbic cortex (PrL/IL), basolateral amygdala (BLA) and choroid plexus (CP) of adult F1 Fischer 344 x Brown Norway (F x BN) rats revealed that *Igf2* mRNA levels were significantly and remarkably higher in all brain regions compared to the peripheral tissues, except for the heart (**Fig 1A and 1B**). The ratio of *Igf2* expression in the brain regions compared to liver, kidney, spleen, and blood was approximately 20–35:1. *Igf2* level in the heart was about 3-fold higher than that of dHC, whereas CP showed the highest expression of all tissues analyzed, with a concentration two orders of magnitude higher than that of the other brain regions examined (**Fig 1A and 1B**). Quantitative western blot analyses confirmed higher IGF2 protein expression in brain regions compared to liver (**Fig 1C**). Thus, in the adult rats, *Igf2* mRNA and/or protein expression is relatively high in the brain compared to many other tissues.

To determine whether the high expression level of *Igf2* in the adult brain regions derives from the maternal or paternal allele, the *Igf2* gene from either F or BN rat brains was sequenced, which identified a polymorphism within a (CA)_n_ repeat in the 3’ untranslated region (UTR) as well as a SNP in the 5’UTR of *Igf2* variant expressed from promoter (P) 2 (**Fig 1D**). Primers flanking the (CA)_n_ repeat region in the 3’UTR used on genomic DNA or RNA produced PCR fragments of 310 base pairs (bp) from BN rats and of 337 bp from F rats, respectively. PCR analysis of the genomic DNA (gDNA) extracted from the F1 hybrid of FxBN confirmed the heterozygous pattern (**Fig 1E**). RT-PCR amplification of RNA obtained from adult F (dam) x BN (sire) rats showed, in agreement with previous reports [1–3], that, except for CP in which the *Igf2* expression is biallelic, the peripheral tissues, including liver, spleen, kidney and blood, express the paternal allele only, confirming maternal *Igf2* imprinting. In contrast, in all the other brain regions analyzed, including dHC, ACC, PrL/IL and BLA, *Igf2* was mainly expressed from the maternal allele, despite low levels of the paternal allele expression (**Fig 1E**). Similar results were obtained with brain tissues extracted from the reciprocal cross, adult BN (dam) x F (sire) rats (**Fig 1E**). No PCR product was observed from the samples run in parallel in the absence of Reverse transcriptase (RT-), excluding the possibility of gDNA contamination (**Fig 1E**).

**Fig 2:**
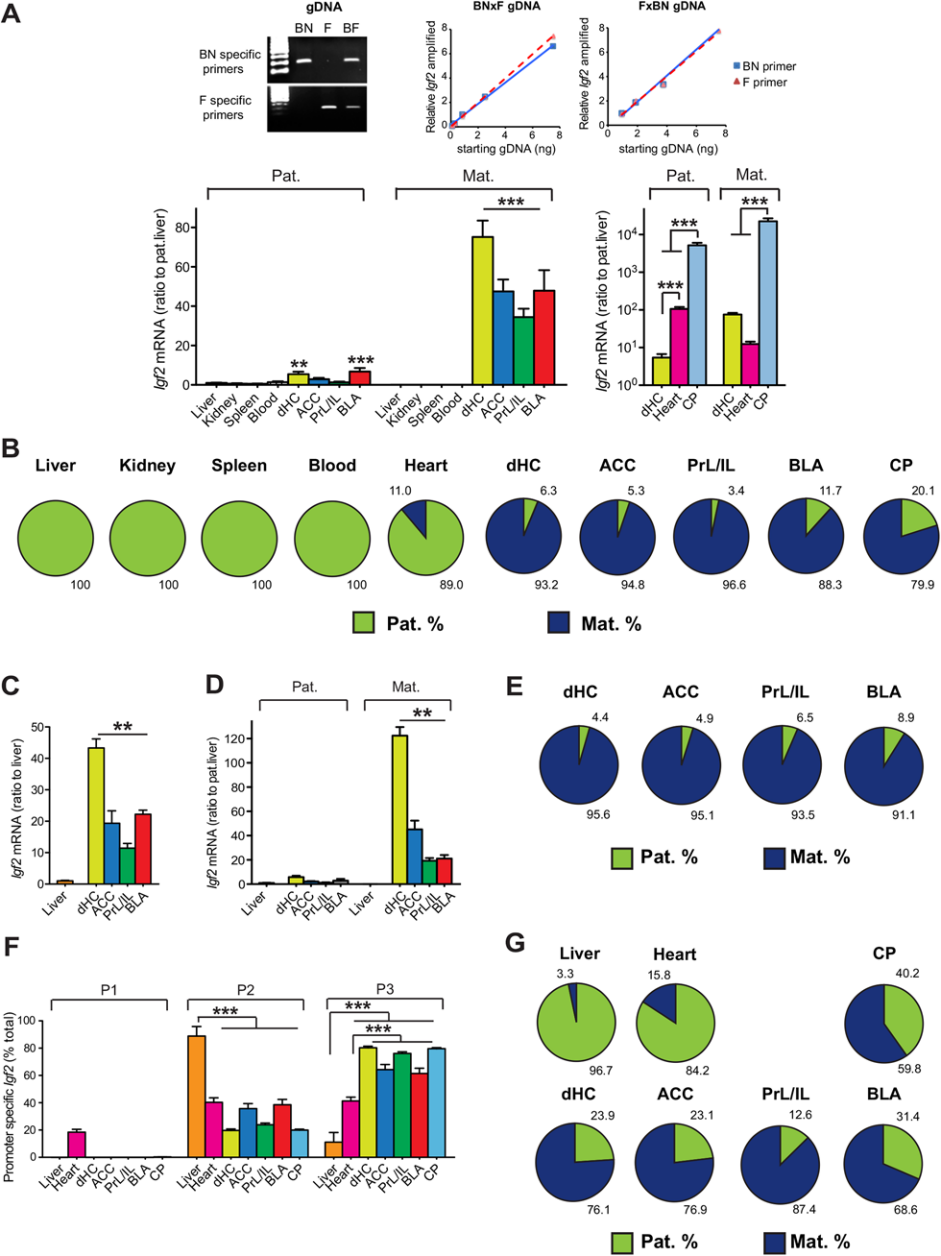
The elevated *Igf2* expression in adult brain regions, compared to peripheral tissues, is mainly due to a dramatic increase in *Igf2* expression from the maternal allele. (A) The top panels demonstrate the specificity and amplification efficiency of BN and F specific primer sets used in the analysis. The bottom panels show the data from quantitative real-time PCR analysis of *Igf2* mRNA expressed from paternal (Pat.) and maternal (Mat.) alleles in various peripheral tissues and brain regions obtained from adult male F1 rats (n = 4–7; F_(19, 112)_ = 25.66, *P* < 0.0001). Data analyses combined F1 offspring from both the F (dam) x BN (sire) cross and the reciprocal BN (dam) x F (sire) cross. *Igf2* mRNA level in each tissue was normalized to the 18S RNA level of that tissue. Data are represented as mean ± SEM of the ratio of *Igf2* mRNA expression relative to the paternal *Igf2* level from liver. **P<0.01, ***P<0.001 (B) Schematic representations of the % expression of *Igf2* mRNA from the paternal or maternal allele in each tissue analyzed from adult male F1 rats. (C) Quantitative real-time PCR analysis of the relative *Igf2* mRNA levels in liver, dHC, ACC, PrL/IL and BLA obtained from adult female F1 rats (n = 6–7; F_(4, 28)_ = 53.31, *P* < 0.0001). In each sample, *Igf2* mRNA level was normalized to the 18S RNA level. Data are expressed as mean ± SEM of the ratio of *Igf2* mRNA in the liver. **P<0.01. (D) Quantitative real-time PCR analysis of *Igf2* mRNA expressed from paternal (Pat.) and maternal (Mat.) alleles in different brain regions of adult female F1 offspring from F (dam) x BN (sire) cross and the reciprocal BN (dam) x F (sire) cross, compared to the *Igf2* level expressed in the liver (n = 6–7; F_(8, 46)_ = 121.8, *P* < 0.0001). *Igf2* mRNA level in each region was normalized to the 18S RNA level in the same region. Data are represented as mean ± SEM of the ratio of *Igf2* mRNA expression relative to the paternal *Igf2* level from liver. **P<0.01. (E) Schematic representations of the % expression of *Igf2* mRNA from the paternal or maternal allele in each adult female tissue analyzed. (F) Quantitative real-time PCR analysis of *Igf2* mRNA expressed from different promoters (P1, P2 and P3) in liver, heart, dHC, ACC, PrL/IL and BLA of adult F1 offspring from F (dam) x BN (sire) cross and the reciprocal BN (dam) x F (sire) cross. Data are presented as mean ± SEM of *Igf2* mRNA expressed from each promoter as percentage of the total *Igf2* mRNA expressed in the region analyzed. (n = 5–7, P2: F_(6, 37)_ = 43.66, *P* < 0.0001; P3: F_(6,37)_ = 50.91, *P* < 0.0001). ***P<0.0001. (G) Schematic representations of the % expression of P2-derived *Igf2* mRNA from the paternal or maternal alleles, analyzed by pyrosequencing (n = 5–7).

Furthermore, qPCR analysis with allele-specific primers was used to quantify maternal *vs*. paternal *Igf2* expression in different tissues of adult male F1 hybrid of F (dam) x BN (sire) and reciprocal cross BN (dam) x F (sire) rats. Results confirmed that *Igf2* was maternally imprinted in liver, kidney, spleen, blood and, even in the heart where it was very highly expressed, *Igf2* was 89% paternal (**Fig 2A and 2B**). In contrast, in all brain regions analyzed, *Igf2* was highly and mainly expressed from the maternal allele. The higher *Igf2* levels in adult brain, compared to peripheral tissues, was mainly due to a dramatic increase in maternal *Igf2* expression, though the paternal allele expression remained at a similar or even higher levels than in peripheral tissues. The preferential maternal expression of *Igf2* in the brain was found to be similar in both F1 genders (**Fig 2 compare B and E).** We concluded that in adult rats, in contrast to peripheral tissues, which show maternal imprinting, *Igf2* is relatively highly expressed in the brain and its expression mostly derives from the maternal allele, consistent with and extending earlier reports by Gregg et al. (2010)[25] (but see DeVeale et al., 2012 [26]), Harper et al. (2014) [27] and Baran et al. (2015) [28].

To examine whether differential promoter usage contributes to the maternal *Igf2* expression in the brain, we analyzed *Igf2* mRNA variants expressed from the three major promoters (P1, P2 and P3) by qPCR. We found that in all adult brain areas investigated, including dHC, ACC, PrL/IL, BLA and CP, *Igf2* was mostly expressed from P3, whereas in peripheral tissues including liver and heart the expression was mostly from for P2 and/or P1 (**Fig 2F**). Further sequencing analysis identified a SNP between BN and F rats in the 5’UTR of *Igf2* mRNA expressed from P2 (**Fig 1D**). We then used allele-specific pyrosequencing to analyze parental allelic expression of *Igf2* from P2 in different tissues. Consistent with our findings obtained with the 3’UTR SNP, *Igf2* expression from P2 was mostly paternal in liver and heart, and mostly maternal in brain regions (**Fig 2G**). Therefore, the differential usage of P2 in different tissues does not seem to explain their distinct allelic expression.

**Fig 3:**
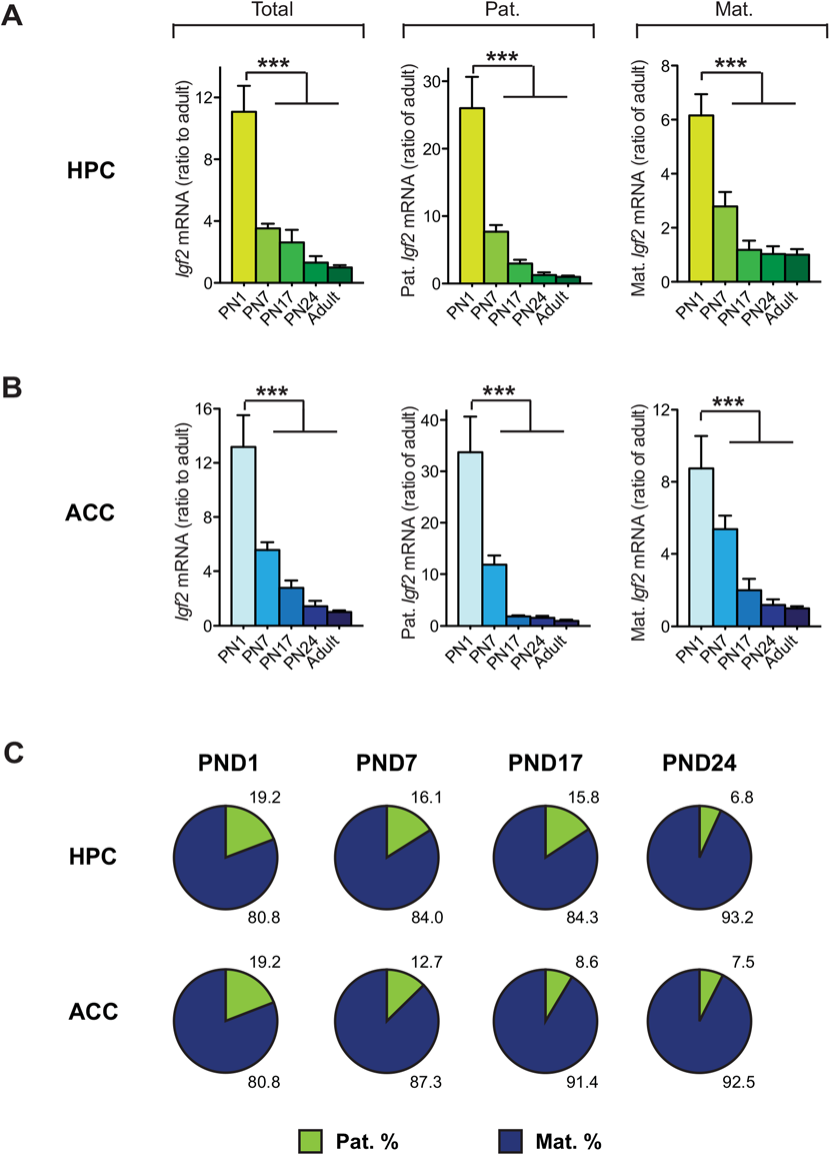
*Igf2* mRNA levels in the brain are down-regulated from both paternal and maternal alleles during postnatal development, with a relatively larger decrease of paternal *Igf2*. (A-B) Quantitative real-time PCR analysis of total *Igf2* level, *Igf2* expressed from paternal (Pat.) and maternal (Mat.) alleles, in the hippocampus (HPC; A) and anterior cingulate cortex (ACC; B), collected from postnatal day (PND) 1, 7, 17 and 24 of F1 rats, compared to *Igf2* levels of adult HPC (n = 5–7; for total *Igf2*: F_(4, 25)_ = 23.06, *P* < 0.0001; for paternal *Igf2*: F_(4, 25)_ = 24.42, *P* < 0.0001; for maternal *Igf2*: F_(4, 25)_ = 21.45, *P* < 0.0001) and ACC (n = 5–9; for total *Igf2*: F_(4, 27)_ = 22.52, *P* < 0.0001, *P* < 0.0001; for paternal *Igf2*: F_(4, 27)_ = 21.32, *P* < 0.0001; for maternal *Igf2*: F_(4, 27)_ = 14.75, *P* < 0.0001). Data analyses combined male and female F1 offspring from both the F (dam) x BN (sire) cross and the reciprocal BN (dam) x F (sire) cross. *Igf2* mRNA level in each region was normalized to the 18S RNA level of the same region. Data are represented as mean ± SEM of the ratio of *Igf2* mRNA levels in developmental HPC and ACC over adult HPC and ACC, respectively. ***P<0.001 (C) Schematic representations of the % expression of *Igf2* mRNA from the paternal or maternal allele in each tissue analyzed.

We next asked whether the ratio of maternal *vs*. paternal *Igf2* allele expression varies over postnatal development. Using qPCR, *Igf2* levels in the hippocampus (HPC) and ACC were quantified at post-natal day (PND) 1, 7, 17, 24 of F (dam) x BN (sire) and BN (dam) x F (sire) F1 offsprings and compared to *Igf2* levels in the same brain regions of adult F (dam) x BN (sire) and BN (dam) x F(sire) F1rats. *Igf2* levels in both HPC and ACC decreased during the course of postnatal development to adult age, with the most dramatic decrease occurring between PND 1 to PND 7 (**Fig 3A and 3B**). This reduction in *Igf2* over the course of development occurred for both alleles, with a significantly larger decrease in the paternal expression (*P*<0.0001). This gradual decrease in the percentage of paternal *Igf2* expression over development (**Fig 3C**) suggests that the dominance in maternal *Igf2* expression is, in part a developmental process. We also conclude that the dominant maternal expression in adult brain, confirmed with 3 independent techniques, is not due to the decrease of *Igf2* expression in adulthood, as the expression in the peripheral tissues where *Igf2* levels are even lower is actually paternal.

Furthermore, despite the high ratio of maternal *Igf2* expression, the brain also shows *Igf2* paternal allele expression at a similar or even higher level than that found in the peripheral tissues. Thus, the reverse pattern of *Igf2* expression in the brain cannot be simply explained by a loss-of-imprinting or reversed (paternal) imprinting of *Igf2*. Possible explanations are that *Igf2* is either paternally imprinted in most cells of the brain but maternally imprinted in a small proportion of them, or that the brain shows imprinting relaxation. The brain includes many cell types, and cell type-specific analysis of allelic expression will provide important information to address this issue. It is also possible that, as suggested by Jouvenot et al. (1999)[29], differential post-transcriptional regulation may contribute to or account for differential parental *Igf2* expression in the brain compared to peripheral tissues. Furthermore, we cannot exclude that species-specific patterns of allele expression regulation occurs. Investigating adult brain *Igf2* allele expression in specific cell populations of different species should help clarify this issue.

**Fig 4:**
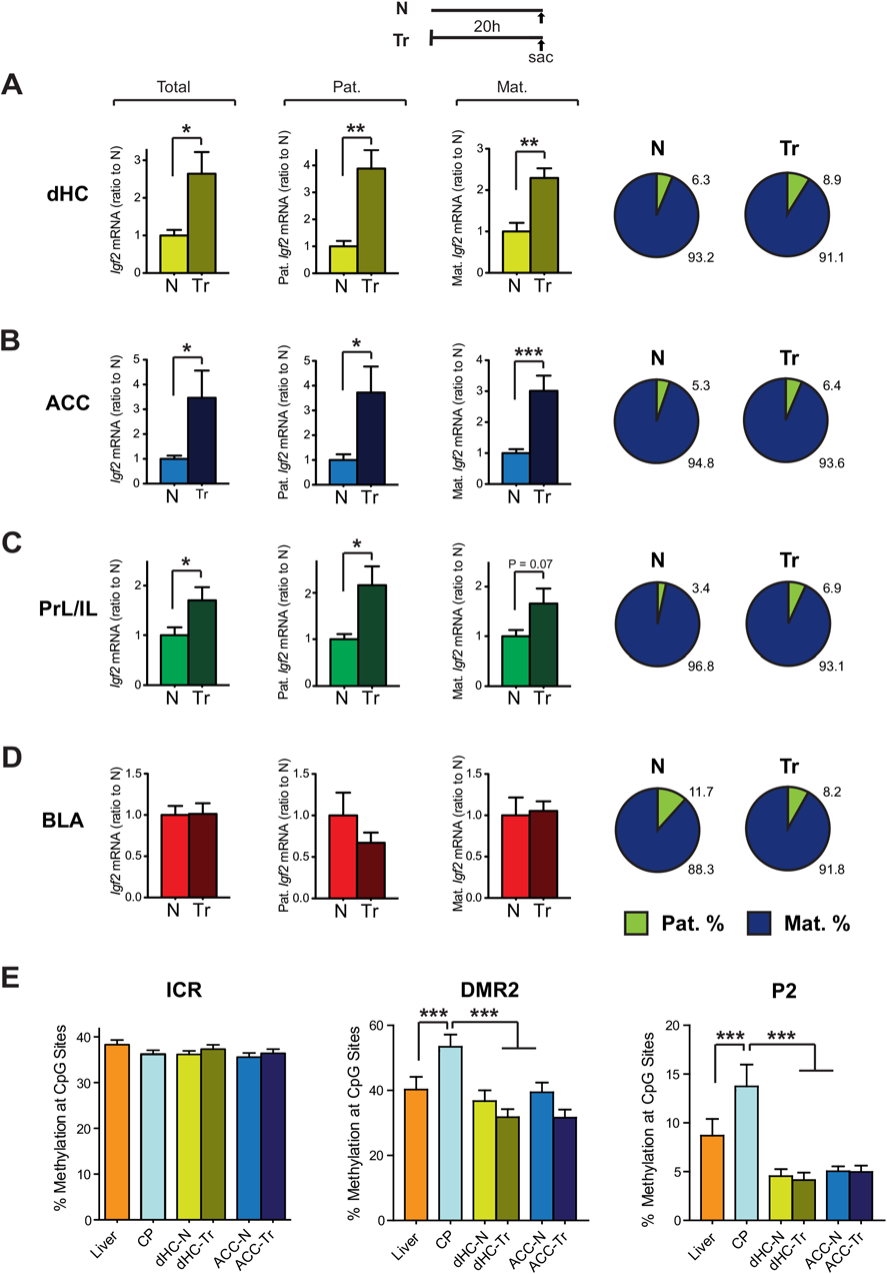
Inhibitory avoidance training results in an increase of *Igf2* mRNA levels in the dHC, ACC, and PrL/IL from both parental alleles at 20hr post-training. Adult male F1 rats were trained in IA, and 20hrs later, brains were collected from both trained (Tr) rats and control naïve (N) rats. Quantitative real-time PCR was used to analyze the total, paternal (Pat.) and maternal (Mat.) *Igf2* mRNA levels in the dHC (A, n = 6–7; for total *Igf2*: t_11_ = 2.96, *P* = 0.013; for paternal *Igf2*: t_11_ = 4.31, *P* = 0.0012; for maternal *Igf2*: t_11_ = 4.12, *P* = 0.0017), ACC (B, n = 7–9; for total *Igf2*: t_14_ = 2.54, *P* = 0.0235; for paternal *Igf2*: t_14_ = 2.87, *P* = 0.0124; for maternal *Igf2*: t_14_ = 4.44, *P* = 0.0006), PrL/IL (C, n = 7; for total *Igf2*: t_12_ = 2.28, *P* = 0.04; for paternal *Igf2*: t_12_ = 2.77, *P* = 0.017; for maternal *Igf2*: t_12_ = 2.02, *P* = 0.066) and BLA (D, n = 6–7; for total *Igf2*: t_11_ = 0.07, *P* = 0.94; for paternal *Igf2*: t_11_ = 1.15, *P* = 0.27; for maternal *Igf2*: t_11_ = 0.23, *P* = 0.82). *Igf2* mRNA level in each sample was normalized to that of the 18S RNA level. Data are presented as mean ± SEM of the ratio of *Igf2* levels in the brain regions taken from trained to naïve rats. Schematic representations of the % expression of *Igf2* mRNA from the paternal or maternal allele in each tissue analyzed. *P<0.05, **P<0.01, ***P<0.001. (E) Analysis of the CpG methylation levels by methylation-sensitive pyrosequencing in *Igf2*/H-19 imprinting control region (ICR), differentially methylation region 2 (DMR2) and P2 promoter of *Igf2* gene in liver, CP, dHC and ACC, as well as in dHC and ACC from naïve (N) *versus* trained (Tr) rats. Data are presented as the mean ± SEM of the percentage of methylation across 15 CpG sites in the ICR region (F_(5, 84)_ = 1.18, *P* = 0.33), 19 CpG sites in the DMR2 region (F_(5, 108)_ = 6.28, *P* < 0.0001), and 11 CpG sites in the P2 region (F_(5, 60)_ = 8.71, *P* < 0.0001). ***P<0.001.

Because we have previously reported that inhibitory avoidance (IA) training in rats leads to an increase in *Igf2* levels in the dHC at 20hr after training, which is required for memory consolidation [16], we next determined if the learning-dependent *Igf2* increase is selective to one allele. Allele specific qPCR analyses of adult F (dam) x BN (sire) and BN (dam) x F (sire) F1 rat brains collected 20 hrs after IA training detected a significant increase of *Igf2* level in the dHC 20 hrs after training (**Fig 4A**), consistent with Chen et al. (2011). This increase derived proportionally from both alleles without significantly changing the ratio of maternal to paternal *Igf2* mRNA expression levels, which remained 91.1% maternal (**Fig 4A**). Similarly, significant proportional biallelic increases in *Igf2* mRNA levels were found in the ACC (**Fig 4B**) and PrL/IL (**Fig 4C**), but no change in *Igf2* levels was detected in the BLA (**Fig 4D**). Thus, the training-induced increase in *Igf2* mRNA expression in several brain regions occurs proportionally from both parental alleles, suggesting that the mechanisms underlying learning-induced *Igf2* expression are independent from those regulating its imprinting status or its expression during development.

*Igf2* imprinting in peripheral tissues is controlled by the differential methylation in the *Igf2*/H19 imprinting control region (ICR). This ICR contains the binding sites for the CCCTC-binding factor (CTCF), which binds to the unmethylated allele and assembles a chromatin insulator that blocks the interaction between the *Igf2* promoter and a downstream enhancer [30]. In rodents, mutations of the CTCF binding sites in the ICR change *Igf2* imprinting status [31–34]. Furthermore, the CTCF binding sites often overlap with those for zinc-finger protein 57 (ZFP57), which are crucial for maintaining the methylation of the paternal *Igf2*/H19 ICR [35–37]. Increased maternal *Igf2* expression might therefore result from hypermethylation of the CpGs around the CTCF/ZFP57 binding sites in the maternal *Igf2*/H19 ICR, which could be revealed as an overall methylation increase. Thus, we tested whether in the brain the methylation level of *Igf2*/H19 ICR around one of the CTCF/ZFP57 binding sites is different than that of the liver, in which *Igf2* is expressed at lower levels and from the paternal allele. CpG methylation was determined in liver, choroid plexus, dHC and ACC of naïve and trained rats by EpigenDx (Hopkinton, MA) using bisulfite treatment and methylation-sensitive pyrosequencing. No significant differences among any of our samples were found (**Fig 4E**), indicating that the increase in maternal *Igf2* expression in the brain is unlikely due to hypermethylation of the maternal allele of *Igf2/H19* ICR. Changes in methylation levels at the differentially methylated regions (DMRs) and promoter regions of *Igf2* have also been associated with tissue-specific parental allele expression, and it has been shown that CTCF binding to the unmethylated CpG in these regions on the maternal allele suppressed *Igf2* expression [30, 35, 38]. We thus extended the DNA methylation analysis to two additional sites, including DMR2 which functions as an enhancer for paternal *Igf2* expression [39] and a region around P2. We found a significant increase in the DNA methylation level in the CP compared to the other tissues analyzed, which correlated with a dramatic and significant increase in *Igf2* expression from both alleles. However, no significant difference in DNA methylation was observed between liver versus the brain regions dHC and ACC (**Fig 4E**). Thus, the increased maternal *Igf2* expression in brain regions cannot simply be explained by changes in DNA methylation level at these sites investigated. Despite these results, we cannot rule out the possibility that allele-specific changes in DNA methylation occur in these or other *Igf2* sequences.

Together, our results suggest that the regulation found in *Igf2* maternal imprinting of peripheral tissues might not be generalized. Perhaps tissue-specific mechanisms of transcriptional regulation that change over development and are superimposed to imprinting control could explain the reciprocal allelic expression in different tissues. Interestingly, a brain-specific enhancer, active on paternal and maternal *Igf2* alleles, has been identified 5’ of the *Igf2/H19* ICR and thus proposed to be insensitive to ICR function [40]. What remains to be understood, and represents a major question in biology, is the significance of these allelic regulations. We speculate that, as also observed with another imprinted gene, *Grb10* [41], a reciprocal parent-of-origin expression may indicate novel hypotheses for imprinted gene function in the brain and evolution.

In conclusion, as the allelic expression of *Igf2* in the adult rat brain does not parallel the maternal imprinting found in peripheral tissues, we suggest that imprinting regulation of genes in the brain needs to be carefully assessed, including a more detailed analysis of tissue-specific imprinting and promoter activity. This may have important implications for evaluating imprinting dysregulations in neuropsychiatric disorders.
